# Single-cell RNA Sequencing Analysis Reveals the Regulatory Functions of Copines Family Genes in Testicular Cancer Progression

**DOI:** 10.2174/0118715303375462250430055914

**Published:** 2025-05-06

**Authors:** Nan Li, Kai Yu, Delun Huang, Xuehong Zhu, Zhong Lin

**Affiliations:** 1 Department of Reproductive Medicine Center, The Reproductive Hospital of Guangxi Zhuang Autonomous Region, Nanning, 530022, China;; 2 Guangxi Key Laboratory of Efficacy Study on Chinese Materia Medica, Guangxi University of Chinese Medicine, Nanning, 530001, China;; 3 Guangxi Key Laboratory of TCM Formulas Theory and Transformation for Damp Diseases, Guangxi University of Chinese Medicine, Nanning, 530001, China;; 4 Department of Physiology, Guangxi University of Chinese Medicine, Nanning, 530004, China

**Keywords:** Testicular cancer, Copines, PI3K-AKT, scRNA-seq, cell cycle regulation, testicular cancer progression

## Abstract

**Aims:**

The aim of this study is to investigate the expression patterns and regulatory functions of Copines family genes in different cellular subpopulations in testicular cancer based on single-cell data and to analyze the regulatory mechanism of Copines family genes in cancer.

**Background:**

Testicular cancer is a frequently diagnosed male tumor. Emerging evidence suggests that Copines family genes are implicated in a variety of cancer phenotypes and cancer progression. Analyzing the expression pattern of Copines family genes in testicular cancer may help improve the treatment efficacy of the cancer.

**Objective:**

This study sought to characterize the expression profiles of Copines family genes in the cellular subpopulations of testicular cancer and to identify key signaling pathways through which they regulate cancer progression.

**Methods:**

Based on single-cell transcriptomic data of testicular cancer, we classified testicular cancer cell subpopulations and analyzed the expressions of Copines family genes in each subpopulation. Cell subpopulations were grouped according to the expression levels of Copines family genes, and differentially expressed Copines family genes between the groups were screened by differential expression analysis. Functional enrichment analysis on the differentially expressed genes (DEGs) was performed with a clusterprofiler package. Functional pathways enriched by the Copines family genes were calculated by AUCell enrichment score. Copy number variation (CNV) analysis was performed using inferCNV to analyze gene mutation patterns across cellular subpopulations, and pseudotime analysis was conducted using Monocle to infer cellular differentiation pathways of cellular subpopulations.

**Results:**

Single-cell clustering identified four major cell subpopulations, namely, NK/T cells, tumor cells, B cells, and macrophages. Notably, the control samples had a relatively small proportion of tumor cells. Further clustering of the tumor cells identified six cell subpopulations, among which multiple Copines genes, especially CPNE1 and CPNE3, showed a high expression. The testicular cancer samples were grouped by the expression patterns of Copines genes, and the DEGs between groups included GNLY, MGP1, CFD2, CCL21, SPARCL13 as well as some other genes involved in the malignant progression of cancer. Pseudotime analysis showed that the upregulated genes were enriched in cell migration and PI3K-Akt pathway, while the downregulated genes were related to immunity. This indicated that the Copines genes regulated the cellular heterogeneity and malignant transformation in testicular cancer.

**Conclusion:**

This study revealed the potential molecular mechanism through which Copines family genes drove the progression of testicular cancer through regulating PI3K-Akt signaling pathway and cell cycle, providing a new target for the development of precision treatment targeting Copines family genes and prognostic assessment of the cancer.

## 1 INTRODUCTION

Cancer persists as a leading cause of mortality across all over the world [1]. Currently, the therapeutic advancements enable approximately 60% of adolescent and young adult cancer survivors to exhibit life expectancies comparable to the general population [2]. Lymphomas and testicular cancer are predominant among reproductive-aged males, with 5-year survival rates surpassing 80-90% [3]. While testicular cancer is considered a rare male malignancy that accounts for only 1% of all cancer cases among men, it is the most frequently diagnosed solid tumor in men aged 15-44 [4]. The majority of cases are germ cell tumors, typically detected in young adulthood [5]. Overall, the current cure rates of testicular cancer reach about 95%, resulting in a growing population of long-term survivors following cancer treatment [6]. Nevertheless, the morbidity and mortality still remain high after implementing standard interventions, such as surgery, radiotherapy, and chemotherapy [7]. Cisplatin-based chemotherapies introduced in the 1970s have markedly improved the clinical outcome, however, their long-term side effects continue to present a substantial challenge for healthcare providers [8-10]. As a result, there is an urgent need to identify molecular drivers of testicular cancer progression to facilitate the development of targeted therapies and refine prognostic strategies.

Copines family proteins are a group of cell membrane-binding proteins, with nine mammalian members (CPNE1-9) implicated in diverse biological processes [11, 12]. Emerging evidence highlights that Copines family members are aberrantly expressed in a variety of cancers and are correlated with malignant behaviors such as tumor cell proliferation, invasion, and metastasis [13, 14]. For example, the expression of CPNE1-3 is commonly upregulated in testicular tissues and is positively correlated with centroblast differentiation to influence cancer malignant progression [15]. Several experimental studies have shown that dysregulation of Copines family may disrupt cell response to growth inhibition and apoptosis, thereby accelerating the growth and spread of testicular cancer cells [16]. This suggested that inhibiting the abnormal activity of Copines family members could block the metastatic phenotype of testicular cancer cells, providing new insights for the clinical treatment of testicular cancer. In the regulatory mechanism of cancer, Copines family members bind to cell membrane through its C2 structural domain and participate in the biological processes, including cell adhesion, cell migration, and signal transduction [17]. However, the specific mechanism of action of Copines family genes in the pathogenesis of testicular cancer [18] and whether they can directly regulate the malignant phenotype of testicular cancer cells remain unclear. Previous studies reported that the Copines family members enhance cell proliferation and migration in osteosarcoma through MAPK pathway, whereas in colorectal cancer, they promote cancer chemoresistance through activating the GLUT1/HK2 cascade [19, 20]. Hence, systematic characterization of the expression patterns of the Copines family in testicular cancer tissues and cell lines may improve our understanding of their role in cancer development, contributing to the discovery of molecular targets for the early detection and treatment of testicular cancer.

Here, we analyzed the role of Copines family genes in testicular cancer progression at single-cell level and classified the cellular subpopulations according to different expression patterns of Copines family genes. In addition, the function and mutation patterns of Copines family genes in testicular cancer progression were comprehensively explored. Overall, the present results provided a new theoretical basis and potential therapeutic targets for the targeted therapy of testicular cancer. The flow chart of the study is shown in Fig. (**1**).

## 2 MATERIALS AND METHODS

### 2.1 Single-cell RNA-seq Data Acquisition and Preprocessing

Data on testicular cancer and its control samples were obtained from GSE197778 in the Gene Expression Omnibus (GEO) website (https://www.ncbi.nlm.nih.gov/geo/). This dataset included transcriptomic profiles of testicular tumor homogenates, pelvic lymph node homogenates, and renal hilus lymph node homogenates, which were all processed using 10x Genomics library preparation. The combination of these three types of samples allowed us to systematically investigate the mechanism of testicular cancer invasion and metastasis.

The scRNA-seq data were pre-processed using the Read10x function of the Seurat package to read the data and retain cells with less than 10% of mitochondrial genes and expressing between 200 and 6000 genes [21, 22]. The NormalizeData function was used for normalizing the data. After downscaled by principal component analysis (PCA), batch effects between samples (max.iter.harmony=20, lambda=0.5) were removed by the harmony package [23, 24]. Next, UMAP downscaling was conducted based on the first 30 principal components (PCs), and then the cell subpopulations were clustered using the FindNeighbors and FindClusters functions [24]. Subsequently, high-expressed genes in each cell subpopulation were screened by the FindAllMarkers, and the DEGs with *p*<0.05 and |logFC| > 1 were further selected using the FindMarkers [25].

### 2.2 Functional Enrichment Analysis

Gene Ontology (GO) analysis of the DEGs was performed using the clusterprofiler package to reveal their biological functions (parameters: keyType = “SYMBOL”, pvalueCutoff = 0.05, qvalueCutoff = 0.1). Correlation between the results of GO and KEGG enrichment analysis across the subgroups was analyzed using simplifyEnrichment package [26, 27].

### 2.3 Calculation of the AUCell Enrichment Scores

The AUCell package was employed to calculate the enrichment scores for Th17 cell differentiation, oocyte meiosis, focal adhesion, PI3K-Akt signaling pathway, Th1 and Th2 cell differentiation, ECM-receptor interaction, and progesterone-mediated oocyte maturation signaling pathway.

### 2.4 CNV Analysis

The count matrix and cell type annotation data of tumor-derived endothelial cells (TECs) and the annotation files of the genes were extracted. B cells served as the control cells to create inferCNV objects using the CreatInfercnvObject function of the inferCNV package. CNV analysis was conducted by applying the infercnv::run function.

### 2.5 Pseudotime Analysis

Pseudotime analysis was performed using Monocle. The DEG (|log2FC|>0.25, min.pct=0.25) between groups were identified using the FindMarkers function. The newCellDataSet was employed to construct cds objects and filter low-quality cells, followed by compressing the data using the DDRTree algorithm *via* the reduceDimension function. The cells were ordered by the orderCells function, and then the plot_cell_trajectory function and plot_genes_in_pseudotime function were applied to plot the trajectory and scatter plots of gene changes with pseudotime.

### 2.6 Statistical Analysis

Differences in continuous variables between two groups were compared using student’s t-test. All the calculations were conducted in R language (version 4.3.1). *P*<0.05 was defined as statistically significant.

## 3 RESULTS

### 3.1 Cell Landscape of Testicular Cancer

**Fig. (2):**
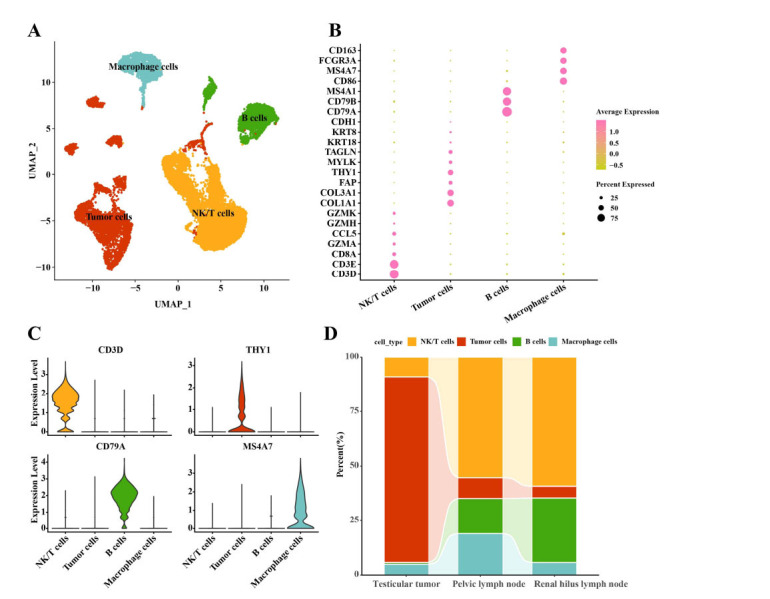
Single-cell mapping of testicular cancer. (**A**) UMAP downscaling plot after clustering annotation of cells in testicular cancer. (**B**) Bubble plots of marker gene expression in cellular subpopulations. (**C**) Violin plots of marker gene expression in cellular subpopulations. (**D**) Proportion of cell subpopulations within testicular cancer samples.

Using the Seurat package, a total of 3627 cells from testicular cancer samples were screened after pre-processing and further classified into four major cell subpopulations, namely, NK/T cells, tumor cells, B cells, and macrophage cells (Fig. **2A**). Specifically, NK/T cells high-expressed CD3D, CD3E, CD8A, GZMA, CCL5, GZMH, GZMK; Tumor cells high-expressed COL1A1, COL3A1, FAP, THY1, MYLK, TAGLN, KRT18, KRT8, CDH1; B cells high-expressed CD79A, CD79B, MS4A1; Macrophages high-expressed CD86, MS4A7, FCGR3A, CD163 (Figs. **2B** and **C**). The proportions of the four cell subpopulations in each sample were quantified, and we found a small number of tumor cells in pelvic lymph nodes and renal hilar lymph nodes, suggesting that Tumor cells in testicular cancer were metastasized. Hence, the tumor cells were the focus in our subsequent analyses (Fig. **2D**).

### 3.2 Single-cell Expression Profiling of Copines Family Genes in the Tumor Cells

**Fig. (3):**
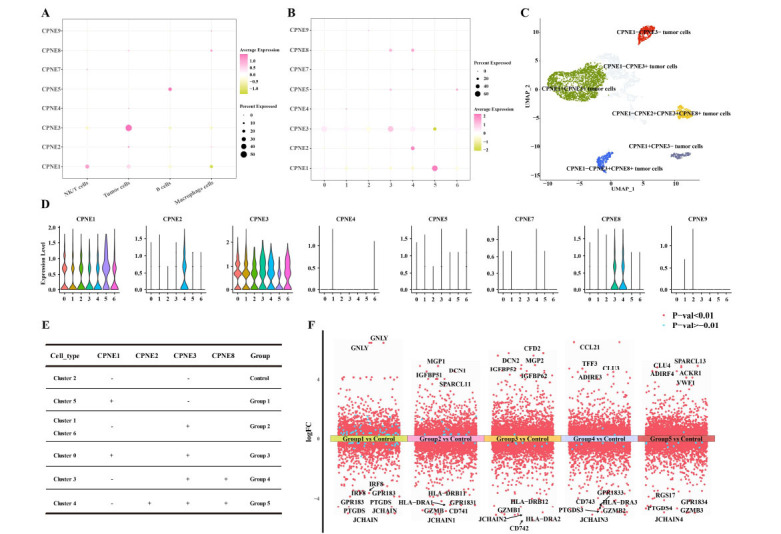
Single-cell mapping of tumor cells. (**A**) Expression of Copines family genes in various cellular subpopulations. (**B**) Expression of Copines family genes in various subpopulations of Tumor cells cells. (**C**) UMAP downscaling map after clustering annotation of Tumor cells. (**D**) Expression of Copines family genes in Tumor cells cell subpopulations in each cell subpopulation. (**E**) Tumor cells subpopulation grouping. (**F**) Differential gene volcano maps for each cellular subpopulation.

Analysis on the expressions of Copines family genes in each cell subpopulation showed that CPNE3 was high-expressed in the tumor cells (Fig. **3A**). Further clustering of the tumor cells identified six subpopulations of the tumor cells, namely CPNE1-CPNE3- Tumor cells, CPNE1+CPNE3- Tumor cells, CPNE1-CPNE3+ Tumor cell, CPNE1+CPNE3+ Tumor cells, CPNE1-CPNE3+CPNE8+ Tumor cells, and CPNE1-CPNE2+CPNE3+CPNE8+Tumor cells (Figs. **3B** and **C**). Multiple Copines genes showed high expression levels in the Tumor cells subpopulations, in particular, CPNE1 and CPNE3 were high-expressed in all the six subpopulations of the Tumor cells (Fig. **3D**). To further explore the association between the expression of Copines family genes and the characteristics of each Tumor cell subpopulation, these subpopulations were further divided into five groups (Group 1-5) and the expressions of the DEGs in the five groups and the control group were determined (Figs. **3E** and **F**). The results demonstrated that the genes associated with malignant progression of cancer, including GNLY [28], MGP1 [29], and CCL21 [30], were significantly upregulated and expressed in the Tumor cell subgroup. These results supported the correlation between Copines family genes and testicular cancer development.

### 3.3 Enrichment Analysis on the DEGs of the Copines Family

**Fig. (4):**
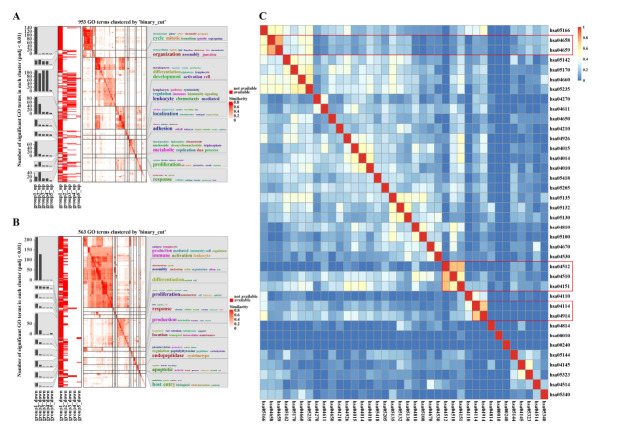
Enrichment analysis of differentially expressed genes. (**A**) Biological process enrichment analysis of differentially up-regulated genes. (**B**) Biological process enrichment analysis of differentially down-regulated genes. (**C**) KEGG enrichment analysis of differential genes.

GO enrichment and KEGG enrichment analyses were performed to investigate the important role of the DEGs across Tumor cell subgroups in the regulation of testicular cancer progression. As shown by the GO analysis, upregulated genes in Group 1 were mainly enriched in biological processes (BPs) such as cell cycle and cell metabolism; in Group 2, they were mainly enriched in regulatory processes such as immune regulation, cell chemotaxis; in Group 3, Group 4 and Group 5, they were mainly enriched in BPs such as activation, differentiation and development of immune cells (Fig. **4A**). The downregulated genes in Group1 were mainly enriched in BPs such as immune activation and immune response; in Group 2, they were mainly enriched in BPs such as immune activation; in Groups 3, 4, and 5, they were mainly enriched in BPs such as chromosome assembly in the nucleus (Fig. **4B**). The KEGG enrichment analysis showed that the differentially expressed Copines family genes were significantly enriched in hsa04658 (Th1 and Th2 cell differentiation), hsa04510 (focal adhesion), hsa04659 (Th17 cell differentiation), hsa04512 (ECM-receptor interaction), hsa04151 (PI3K-Akt signaling pathway), hsa04914 (progesterone-mediated oocyte maturation) and hsa04114 (oocyte meiosis) pathways (Fig. **4C**).

### 3.4 AUCell Enrichment Analysis

**Fig. (5):**
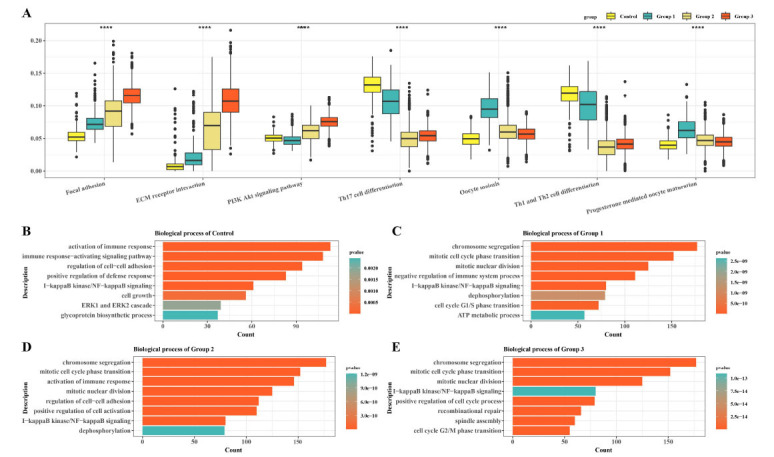
AUCell enrichment analysis. (**A**) AUCell enrichment analysis. **** *p* <0.0001. (**B**) Biofunctional enrichment analysis of highly expressed genes in the Control group. (**C**) Biofunctional enrichment analysis of highly expressed genes in Group 1 group. (**D**) Biofunctional enrichment analysis of highly expressed genes in Group 2 group. (**E**) Biofunctional enrichment analysis of highly expressed genes in Group 3 group.

We further performed AUCell enrichment analysis on the KEGG signaling pathways obtained from the above analysis. The results showed that the enrichment score of PI3K Akt signaling pathway, ECM receptor interaction, and focal adhesion were elevated, while that of Th17 cell differentiation, Th1 and Th2 cell differentiation signaling pathway scores was gradually reduced (Fig. **5A**). To further differentiate the functions among cell subpopulations, we performed GO analysis in BP terms on high-expressed genes in Control, Group 1, Group 2, and Group 3. The results showed that the Control group was mainly enriched in signaling pathways such as activation of immune response, inflammation and signaling (Fig. **5B**), and that Group 1, Group 2 and Group 3 were enriched in almost the same BPs, including mitotic cell cycle phase transition, chromosome segregation, dephosphorylation, mitotic nuclear division, and positive regulation of cycle process (Figs. **5C**-**E**).

### 3.5 CNV Differences in Copines Family Genes Across the Subgroups of Tumor Cells

**Fig. (6):**
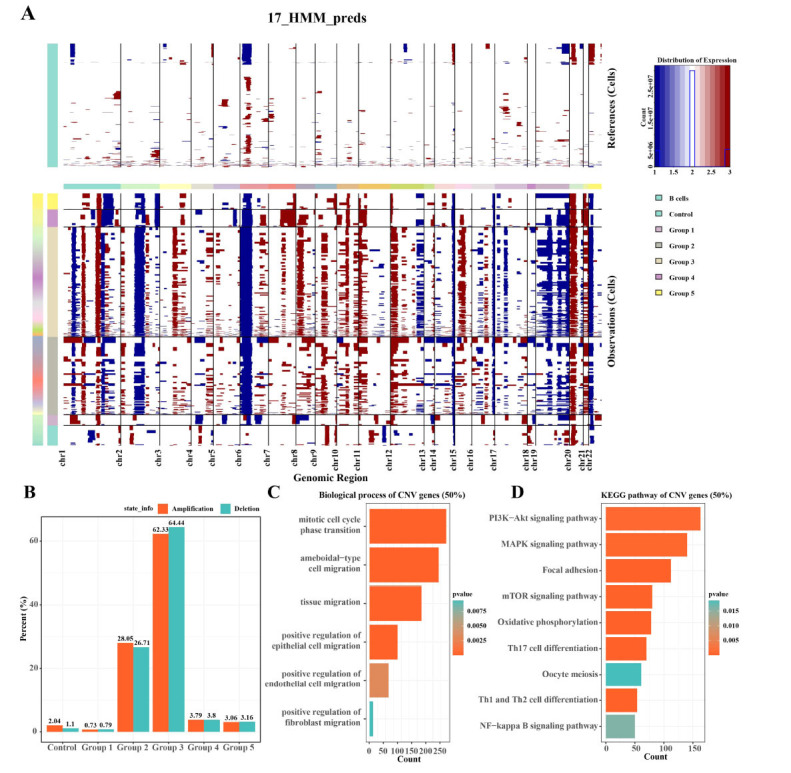
CNV analysis. (**A**) CNV analysis between subgroups with different Copines family gene expression patterns. (**B**) Proportion of variation in CNV genes in each subgroup. (**C**) Biofunctional enrichment analysis of CNV genes. (**D**) KEGG enrichment analysis of CNV genes.

Though the functions between Control, Group 1, Group 2, and Group 3 were almost the same, the results of AUCell enrichment analysis showed progressively higher enrichment scores for PI3K Akt signaling pathway, focal adhesion, and ECM receptor interaction. Hence, we hypothesized that these genes had genomic differences. For this reason, we analyzed CNV variants in each cell group, with B cells as a reference. The results showed that the variant genes were almost the same between the Control and Group 1, while highly variant genes existed in Group 2, Group 3, Group 4, and Group 5 (Fig. **6A**). Next, we quantified the proportion of the variant genes in each group, and it was observed that genes with amplification and deletion accounted for a large percentage (Fig. **6B**). BP analysis on these variant genes demonstrated that they were mainly enriched in cell cycle and cell migration processes (Fig. **6C**). As shown by KEGG enrichment analysis, these genes were mainly enriched in NF-Kappa B signaling pathway, PI3K-Akt signaling pathway, MAPK signaling pathway, *etc*. (Fig. **6D**). Moreover, the variant genes in the PI3K-Akt signaling pathway had the highest degree of gene variation in Group 2 and Group 3 (Fig. **S1**).

### 3.6 Pseudotime Analysis of the Tumor Cells

**Fig. (7):**
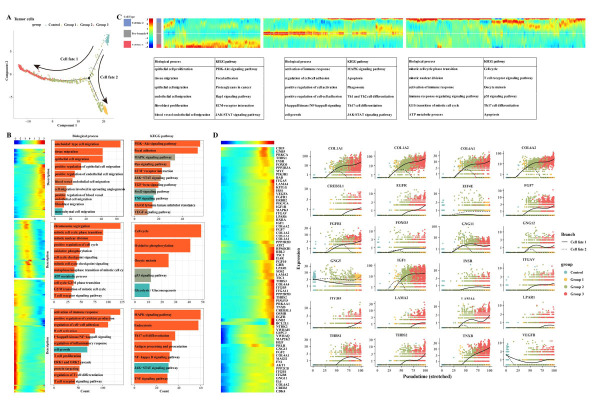
Pseudotime analysis of tumor cells. (**A**) Pseudotime trajectory. (**B**) Pseudotime heatmap. (**C**) Pseudotemporal branching heatmap. (**D**) PI3K-Akt signaling pathway gene expression over time.

To investigate the metastasis mechanism of testicular cancer, we performed Pseudotime analysis on Control, Group 1, Group 2, and Group 3. The results showed that Control eventually developed into Group 1 and Group 3, while Group 2 was in the intermediate process of development (Fig. **7A**). Next, as shown by the analysis on the gene changes during cancer development, gradually upregulated genes were mainly enriched in signaling pathways such as cell migration and PI3k-Akt, while gradually downregulated genes were mainly enriched in processes such as immunity and signaling pathways such as MAPK. Some irregularly changing genes were mainly enriched in BPs, such as cell cycle and signaling pathways, such as oxidative phosphorylation (Fig. **7B**). Similarly, Fig. (**7C**) demonstrated the gene expression changes and functional enrichment features on different branches (Cell fate 1 and Cell fate 2) in the proposed temporal trajectory of tumor cells. The results showed that cells on Cell fate 1 direction were mainly enriched in PI3K-Akt signaling pathway and cell migration and proliferation, suggesting that they had stronger invasive metastatic ability. The Cell fate 2 branch, on the other hand, was enriched in pathways such as cell cycle and activation of immune response. In addition, cells in the Pre-branch were enriched in the MAPK signaling pathway and apoptosis, which revealed that testicular cancer cells are functionally heterogeneous during development and evolution and may receive regulation by multiple signaling pathways. We further evaluated the genes in the PI3K-Akt signaling pathway during cell development, and the results showed that these genes were gradually elevated during cell development (Fig. **7D**). Moreover, the positions of these genes in the signaling pathway are shown in Fig. (**S2**).

## 4 DISCUSSION

The scRNA-seq technique has revolutionized our ability to systematically characterize the cellular environment of tumors and to reveal cell biology, disease origins, and responses to medication [31]. Copines family genes, characterized by their widespread expression, are a recently identified group of proteins that bind to phospholipids. Their functions depend on Ca^2+^ and exhibit structural conservation through evolution [32]. Copines family comprises nine distinct members [17]. While the specific functions and biological significance of Copines are still not fully understood, research suggests that they are involved in various signaling pathways related to tumor formation and development [11]. Here, we performed systematic scRNA-seq analysis to reveal the expressions of Copines family genes in testicular cancer at the single-cell level and to explore the regulatory functions of key Copines family genes.

CPNE1 is an oncogene in tumors, and high-expressed CPNE1 could enhance the growth and metastatic phenotypes of lung and prostate cancers [33, 34]. Silencing CPNE1 significantly inhibits the proliferation and metastasis of osteosarcoma cells. Current studies on the regulatory mechanism of CPNE1 are mainly focused on its regulatory role of glucose-6-phosphate in glucose homeostasis and cellular energy metabolism [19, 35]. High-expressed CPNE3 is involved in tumor cell proliferation and metastasis and promotes a poor prognosis in a variety of cancers, including breast, prostate, and ovarian cancers [36]. CPNE3 exhibits kinase activity, phosphorylates Hl histones, activates downstream signaling pathways, and enhances tumor proliferation and metastasis [37, 38]. Mechanistically, CPNE3 regulates the activation and transduction of multiple signaling pathways including FAK and ErbB2, and affects cancer cell invasion, migration, and proliferation activity [39, 40]. In this study, we analyzed the expressions of Copines family genes (CPNE1-9) in testicular cancer tumor cells, and found significantly high expressions of CPNE1 and CPNE3. This indicated that the two genes played similar pro-cancer roles in the malignant progression of testicular cancer, providing new potential targets for the molecular intervention therapy of testicular cancer.

Testicular cancer samples were grouped according to the expression patterns of Copines genes, and the DEGs between the groups were GNLY, MGP1, CCL21, SPARCL13 and some other genes linked to the malignant progression of the cancer. It has been observed that colorectal cancer samples with a high expression of GNLY have remarkably high heterogeneity of immune cells in the immune infiltration microenvironment compared to the low-expression group. In particular, Tregs have a high infiltration and are related to a malignant phenotype of colorectal cancer cells [41]. MGP1 protein, on the other hand, mainly affects the progression of breast cancer by promoting aberrant angiogenesis of the tumor cells. However, the regulation of its functions differs according to different tumor types [42]. For example, the expression of MGP-like genes is negatively associated with progression and metastatic phenotype in renal and prostate cancers but is positively associated with a poor prognosis in breast cancer and glioblastoma [43, 44]. CCL21, produced primarily by lymphatic endothelial cells, encodes a chemokine ligand. The interaction of CCL21 with dendritic cell surface receptor CCR7 is a major cause of cancer cell migration to lymphoid tissues [45]. CCL21 has been shown to regulate the production of the angiogenic factor VEGF, which controls vascular malformations and chemoresistance of cancer cells [46]. SPARCL family proteins are novel prognostic biomarkers in colorectal cancer and can influence cancer progression by modulating the tumor immune infiltration microenvironment [47]. Mechanistically, SPARCL mediates cancer immune escape by interacting with PI3K-Akt, cGMP-PKG, and some other related pathways, thereby regulating the malignant phenotype of cancer cells [48]. Copines family genes may directly regulate the malignant phenotype of testicular cancer cells through synergistic activation of the genes related to malignant progression of cancers, providing a solid basis for improving the clinical treatments for the cancer.

The subgroups of Tumor cells in this study were mainly enriched in cell cycle regulation, PI3K-Akt and immune response-related signaling pathways. Pseudotime Analysis revealed an inter-translational relationship between the subgroups of Copines family genes. According to the existing studies, the regulation mechanism of Copines family genes on cancer is closely related to cancer types, for example, they regulate cancer cell proliferation and apoptosis by activating the PI3K/AKT signaling pathway in human glioblastoma [49]. This could explain the differential enrichment of the Copines family genes in the PI3K-Akt pathway between the Tumor cell subgroups in this study. In cell cycle regulation, CPNE3 can activate FAK signaling pathway through interacting with RACK1 to promote cancer cell proliferation, migration and invasion [40]. FAK signaling pathway is closely associated with cell cycle regulation, especially in regulating G2/M phase transition and promoting the interaction between extracellular matrix and intracellular cytoskeletal proteins to enhance the metastatic phenotype [50]. In immune regulation, high expressions of Copines family genes are normally positively correlated with the infiltration of immune cells such as regulatory T cells, CD8^+^ T cells, and plasma cells, potentially activating cellular markers for immune escape such as CTLA4, PDCD1, and LAG3 [51]. Hence, Copines family genes may regulate PI3K-Akt signaling pathway, cell cycle and immunoregulatory pathways to affect the differentiation of testicular cancer cells, thereby contributing to the heterogeneity of testicular cancer.

Nevertheless, certain limitations still existed in the current study. Firstly, all the data were sourced from the GEO database (GSE197778) and the reproducibility of the results was not verified by independent samples or experiments. In the future, testicular cancer cell lines and clinical tissue samples will be employed to validate the expression patterns of Copines genes (CPNE1 and CPNE3). Secondly, this study predicted that Copines regulated the PI3K-Akt pathway only by bioinformatics analysis; however, the specific molecular interaction mechanism remained unexplored. For this reason, co-immunoprecipitation will be utilized to identify the binding proteins of Copines and to verify the effects of Copines on downstream pathways (*e.g.* AKT phosphorylation). Finally, single-cell data cannot fully capture the dynamic roles of Copines during tumor evolution. Therefore, future studies will perform single-cell sequencing of paired pre- and post-treatment samples from the same patients to track temporal changes in the expression of Copines family gens. Additionally, CRISPR screening will also be employed to systematically characterize the functional contributions of Copines family genes to the drug resistance in testicular cancer.

## CONCLUSION

In conclusion, comprehensive analyses based on single- cell transcriptomic data in testicular cancer and control samples revealed that the Tumor cells in the testicular cancer tissues had migratory potential, and that multiple Copines family genes were significantly high-expressed in the subpopulations of the Tumor cells. Testicular cancer samples were categorized into subgroups based on the expression patterns of Copines family genes, and functional differences in cell cycle, PI3K-AKT, and immunoregulatory pathways were observed among the subgroups. These findings provided new perspectives for the development of precision treatment and prognostic assessment through targeting Copines family genes.

## Figures and Tables

**Fig. (1):**
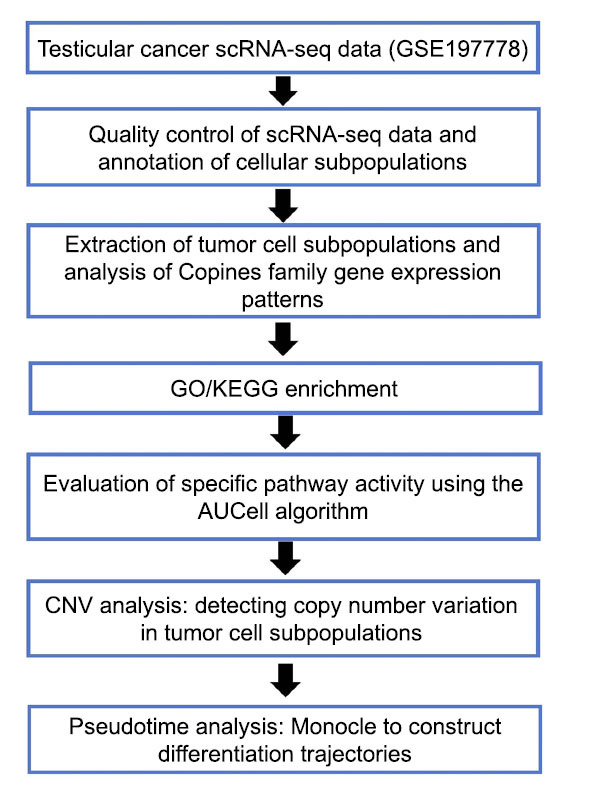
Research flowchart.

## Data Availability

The datasets generated and/or analyzed during the current study are available in the (GSE197778) repository, (https://www.ncbi.nlm.nih.gov/geo/query/acc.cgi?acc=GSE197 778).

## LIST OF ABBREVIATIONS

CNV: Copy Number Variation
DEGs: Differentially Expressed Genes
GEO: Gene Expression Omnibus
GO: Gene Ontology
KEGG: Kyoto Encyclopedia of Genes and Genomes
scRNA-seq: Single-cell Cell Sequencing
TEC: Tumor Endothelial Cell
CPNE: Copines Family Genes
ECM: Extracellular Matrix

## References

[1] Nierengarten M.B. (2024). Cancer Statistics 2024: Deaths drop, incidences increase, prevention needed.. Cancer.

[2] Pallotti F., Petrozzi A., Cargnelutti F., Radicioni A.F., Lenzi A., Paoli D., Lombardo F. (2019). Long-term follow up of the erectile function of testicular cancer survivors.. Front. Endocrinol..

[3] Clasen S.C., Fung C., Sesso H.D., Travis L.B. (2023). Cardiovascular risks in testicular cancer: Assessment, prevention, and treatment.. Curr. Oncol. Rep..

[4] Siegel R.L., Kratzer T.B., Giaquinto A.N., Sung H., Jemal A. (2025). Cancer statistics, 2025.. CA Cancer J. Clin..

[5] Mai P.L., Chen B.E., Tucker K., Friedlander M., Phillips K.A., Hogg D., Jewett M.A.S., Bodrogi I., Geczi L., Olah E., Heimdal K., Fosså S.D., Nathanson K.L., Korde L., Easton D.F., Dudakia D., Huddart R., Stratton M.R., Bishop D.T., Rapley E.A., Greene M.H. (2009). Younger age-at-diagnosis for familial malignant testicular germ cell tumor.. Fam. Cancer.

[6] Khan M.R., Sheehan P.K., Bazin A., Leonard C., Aleem U., Corrigan L., McDermott R. (2024). Late side effects of testicular cancer and treatment: A comprehensive review.. Discov. Oncol..

[7] Sonkin D., Thomas A., Teicher B.A. (2024). Cancer treatments: Past, present, and future.. Cancer Genet..

[8] Kerns S.L., Fung C., Fossa S.D., Dinh P.C., Monahan P., Sesso H.D., Frisina R.D., Feldman D.R., Hamilton R.J., Vaughn D., Martin N., Huddart R., Kollmannsberger C., Sahasrabudhe D., Ardeshir-Rouhani-Fard S., Einhorn L., Travis L.B. (2020). Relationship of cisplatin-related adverse health outcomes with disability and unemployment among testicular cancer survivors.. JNCI Cancer Spectr..

[9] Kerns S.L., Fung C., Monahan P.O., Ardeshir-Rouhani-Fard S., Abu Zaid M.I., Williams A.M., Stump T.E., Sesso H.D., Feldman D.R., Hamilton R.J., Vaughn D.J., Beard C., Huddart R.A., Kim J., Kollmannsberger C., Sahasrabudhe D.M., Cook R., Fossa S.D., Einhorn L.H., Travis L.B., Platinum Study Group (2018). Cumulative burden of morbidity among testicular cancer survivors after standard cisplatin-based chemotherapy: A multi-institutional study.. J. Clin. Oncol..

[10] Einhorn L.H., Donohue J. (1977). Cis-diamminedichloroplatinum, vinblastine, and bleomycin combination chemotherapy in disseminated testicular cancer.. Ann. Intern. Med..

[11] Tang H., Pang P., Qin Z., Zhao Z., Wu Q., Song S., Li F. (2021). The CPNE family and their role in cancers.. Front. Genet..

[12] Vozy A., Coutzac C. (2016). Colitis induced by immune checkpoint inhibitors: Anti-CTLA-4 antibodies and anti-PD-1/PDL-1 antibodies.. Oncologie.

[13] Ding X., Jin Y., Wu Y., Wu Y., Wu H., Xiong L., Song X., Liu S., Fan W., Fan M. (2008). Localization and cellular distribution of CPNE5 in embryonic mouse brain.. Brain Res..

[14] Li Y., Li L., Liu H., Zhou T. (2022). CPNE1 silencing inhibits cell proliferation and accelerates apoptosis in human gastric cancer.. EUR. J. Pharm. Sci..

[15] Cowland J.B., Carter D., Bjerregaard M.D., Johnsen A.H., Borregaard N., Lollike K. (2003). Tissue expression of copines and isolation of copines I and III from the cytosol of human neutrophils.. J. Leukoc. Biol..

[16] Wang A., Yang W., Li Y., Zhang Y., Zhou J., Zhang R., Zhang W., Zhu J., Zeng Y., Liu Z., Huang J. (2022). CPNE1 promotes non-small cell lung cancer progression by interacting with RACK1 *via* the MET signaling pathway.. Cell Commun. Signal..

[17] Khvotchev M., Soloviev M. (2024). Copines, a family of calcium sensor proteins and their role in brain function.. Biomolecules.

[18] Cheng L., Albers P., Berney D.M., Feldman D.R., Daugaard G., Gilligan T., Looijenga L.H.J. (2018). Testicular cancer.. Nat. Rev. Dis. Primers.

[19] Jiang Z., Jiang J., Zhao B., Yang H., Wang Y., Guo S., Deng Y., Lu D., Ma T., Wang H., Wang J. (2018). CPNE1 silencing inhibits the proliferation, invasion and migration of human osteosarcoma cells.. Oncol. Rep..

[20] Wang Y., Pan S., He X., Wang Y., Huang H., Chen J., Zhang Y., Zhang Z., Qin X. (2021). CPNE1 enhances colorectal cancer cell growth, glycolysis, and drug resistance through regulating the AKT-GLUT1/HK2 pathway.. OncoTargets Ther..

[21] Stuart T., Butler A., Hoffman P., Hafemeister C., Papalexi E., Mauck W.M., Hao Y., Stoeckius M., Smibert P., Satija R. (2019). Comprehensive integration of single-cell data.. Cell.

[22] Shahrajabian M.H., Sun W., Survey on Multi-omics, and Multi-omics Data Analysis, Integration and Application (2023). Survey on multi-omics, and multi-omics data analysis, integration and application.. Curr. Pharm. Anal..

[23] Korsunsky I., Millard N., Fan J., Slowikowski K., Zhang F., Wei K., Baglaenko Y., Brenner M., Loh P., Raychaudhuri S. (2019). Fast, sensitive and accurate integration of single-cell data with Harmony.. Nat. Methods.

[24] Zulibiya A., Wen J., Yu H., Chen X., Xu L., Ma X., Zhang B. (2023). Single-cell RNA sequencing reveals potential for endothelial- to-mesenchymal transition in tetralogy of fallot.. Congenit. Heart Dis..

[25] Xu X., Huang Y., Han X. (2024). Single-nucleus RNA sequencing reveals cardiac macrophage landscape in hypoplastic left heart syndrome.. Congenit. Heart Dis..

[26] Gu Z., Hübschmann D. (2023). *SimplifyEnrichment* : A bioconductor package for clustering and visualizing functional enrichment results.. Genomics Proteomics Bioinformatics.

[27] Guo Y., Liu X., Xu Q., Zhou X., Liu J., Xu Y., Lu Y., Liu H. (2024). Revealing the role of honokiol in human glioma cells by RNA-seq analysis.. Biocell.

[28] Milovanović J., Todorović-Raković N., Vujasinović T., Greenman J., Mandušić V., Radulovic M. (2022). Can granulysin provide prognostic value in primary breast cancer?. Pathol. Res. Pract..

[29] Muneer R.S., Gray P.N. (1979). Alteration of human breast tumor cell membrane functions by chromosome-mediated gene transfer.. J. Supramol. Struct..

[30] del Molino del Barrio I., Meeson A., Cooke K., Malki M.I., Barron-Millar B., Kirby J.A., Ali S. (2021). Contribution of heparan sulphate binding in CCL21-mediated migration of breast cancer cells.. Cancers.

[31] Liu H., Dong A., Rasteh A.M., Wang P., Weng J. (2024). Identification of the novel exhausted T cell CD8^+^ markers in breast cancer.. Sci. Rep..

[32] Creutz C.E., Tomsig J.L., Snyder S.L., Gautier M.C., Skouri F., Beisson J., Cohen J. (1998). The copines, a novel class of C2 domain-containing, calcium-dependent, phospholipid-binding proteins conserved from Paramecium to humans.. J. Biol. Chem..

[33] Liu S., Tang H., Zhu J., Ding H., Zeng Y., Du W., Ding Z., Song P., Zhang Y., Liu Z., Huang J.A. (2018). High expression of Copine-1 promotes cell growth and metastasis in human lung adenocarcinoma.. Int. J. Oncol..

[34] Liang J., Zhang J., Ruan J., Mi Y., Hu Q., Wang Z., Wei B. (2017). CPNE1 is a useful prognostic marker and is associated with TNF receptor-associated factor 2 (TRAF2) expression in prostate cancer.. Med. Sci. Monit..

[35] Park N., Yoo J.C., Lee Y.S., Choi H.Y., Hong S.G., Hwang E.M., Park J.Y. (2014). Copine1 C2 domains have a critical calcium-independent role in the neuronal differentiation of hippocampal progenitor HiB5 cells.. Biochem. Biophys. Res. Commun..

[36] Sun B., Li Y., Zhou Y., Ng T.K., Zhao C., Gan Q., Gu X., Xiang J. (2019). Circulating exosomal CPNE3 as a diagnostic and prognostic biomarker for colorectal cancer.. J. Cell. Physiol..

[37] Thomas G., Jacobs K.B., Yeager M., Kraft P., Wacholder S., Orr N., Yu K., Chatterjee N., Welch R., Hutchinson A., Crenshaw A., Cancel-Tassin G., Staats B.J., Wang Z., Gonzalez-Bosquet J., Fang J., Deng X., Berndt S.I., Calle E.E., Feigelson H.S., Thun M.J., Rodriguez C., Albanes D., Virtamo J., Weinstein S., Schumacher F.R., Giovannucci E., Willett W.C., Cussenot O., Valeri A., Andriole G.L., Crawford E.D., Tucker M., Gerhard D.S., Fraumeni J.F., Hoover R., Hayes R.B., Hunter D.J., Chanock S.J. (2008). Multiple loci identified in a genome-wide association study of prostate cancer.. Nat. Genet..

[38] Mo W., Zhang J., Li X., Meng D., Gao Y., Yang S., Wan X., Zhou C., Guo F., Huang Y., Amente S., Avvedimento E.V., Xie Y., Li Y. (2013). Identification of novel AR-targeted microRNAs mediating androgen signalling through critical pathways to regulate cell viability in prostate cancer.. PLoS One.

[39] Choi H.Y., Park N., Na J.B., Ko E.S., Park J.Y., Yoo J.C. (2016). Direct binding of Copine3 with Jab1 activates downstream ErbB2 signaling and motility in SKBr3 breast cancer cells.. Oncol. Rep..

[40] Lin H., Zhang X., Liao L., Yu T., Li J., Pan H., Liu L., Kong H., Sun L., Yan M., Yao M. (2018). CPNE3 promotes migration and invasion in non-small cell lung cancer by interacting with RACK1 *via* FAK signaling activation.. J. Cancer.

[41] Zhang L., Yu X., Zheng L., Zhang Y., Li Y., Fang Q., Gao R., Kang B., Zhang Q., Huang J.Y., Konno H., Guo X., Ye Y., Gao S., Wang S., Hu X., Ren X., Shen Z., Ouyang W., Zhang Z. (2018). Lineage tracking reveals dynamic relationships of T cells in colorectal cancer.. Nature.

[42] Yoshimura K., Takeuchi K., Nagasaki K., Ogishima S., Tanaka H., Iwase T., Akiyama F., Kuroda Y., Miki Y. (2009). Prognostic value of matrix Gla protein in breast cancer.. Mol. Med. Rep..

[43] Levedakou E.N., Strohmeyer T.G., Effert P.J., Liu E.T. (1992). Expression of the matrix Gla protein in urogenital malignancies.. Int. J. Cancer.

[44] Mertsch S., Schurgers L.J., Weber K., Paulus W., Senner V. (2009). Matrix gla protein (MGP): An overexpressed and migration-promoting mesenchymal component in glioblastoma.. BMC Cancer.

[45] Han L., Zhang L. (2023). CCL21/CCR7 axis as a therapeutic target for autoimmune diseases.. Int. Immunopharmacol..

[46] Geraldo L.H., Garcia C., Xu Y., Leser F.S., Grimaldi I., de Camargo Magalhães E.S., Dejaegher J., Solie L., Pereira C.M., Correia A.H., De Vleeschouwer S., Tavitian B., Canedo N.H.S., Mathivet T., Thomas J.L., Eichmann A., Lima F.R.S. (2023). CCL21-CCR7 signaling promotes microglia/macrophage recruitment and chemotherapy resistance in glioblastoma.. Cell. Mol. Life Sci..

[47] Zhang H.P., Wu J., Liu Z.F., Gao J.W., Li S.Y. (2022). SPARCL1 is a novel prognostic biomarker and correlates with tumor microenvironment in colorectal cancer.. BioMed Res. Int..

[48] Wang Y., Li W., Jin X., Jiang X., Guo S., Xu F., Su X., Wang G., Zhao Z., Gu X. (2021). Identification of prognostic immune-related gene signature associated with tumor microenvironment of colorectal cancer.. BMC Cancer.

[49] Zhang D., Wang X., Wang X., Wang Z., Ma S., Zhang C., Li S., Jia W. (2021). CPNE3 regulates the cell proliferation and apoptosis in human Glioblastoma *via* the activation of PI3K/AKT signaling pathway.. J. Cancer.

[50] Zhang X., Yao Z., Xue Z., Wang S., Liu X., Hu Y., Zhang Y., Wang J., Li X., Chen A. (2022). Resibufogenin targets the ATP1A1 signaling cascade to induce G2/M phase arrest and inhibit invasion in glioma.. Front. Pharmacol..

[51] Zhou H., He Y., Huang Y., Li R., Zhang H., Xia X., Xiong H. (2023). Comprehensive analysis of prognostic value, immune implication and biological function of CPNE1 in clear cell renal cell carcinoma.. Front. Cell Dev. Biol..

