# Supplementary material for: Single-cell RNA Sequencing Analysis Reveals the Regulatory Functions of Copines Family Genes in Testicular Cancer Progression
Source: Endocr Metab Immune Disord Drug Targets. 2025 May 6;26:E18715303375462. doi: 10.2174/0118715303375462250430055914 (PMC13334245; doi:10.2174/0118715303375462250430055914)
Supplement: Supplementary file 1 [file EMIDDT-26-E18715303375462_SD1.pdf]

Supplementary Material

Single-cell RNA Sequencing Analysis Reveals the Regulatory Functions of Copines Family Genes in Testicular Cancer Progression

Nan Li[1,#] , Kai Yu[2,3,#], Delun Huang[4], Xuehong Zhu[1,*] and Zhong Lin[1,*]

[1]Department of Reproductive Medicine Center, The Reproductive Hospital of Guangxi Zhuang Autonomous Region, Nanning, 530022, China; [2]Guangxi Key Laboratory of Efficacy Study on Chinese Materia Medica, Guangxi University of Chinese Medicine, Nanning, 530001, China; [3]Guangxi Key Laboratory of TCM Formulas Theory and Transformation for Damp Diseases, Guangxi University of Chinese Medicine, Nanning, 530001, China; [4]Department of Physiology, Guangxi University of Chinese Medicine, Nanning, 530004, China

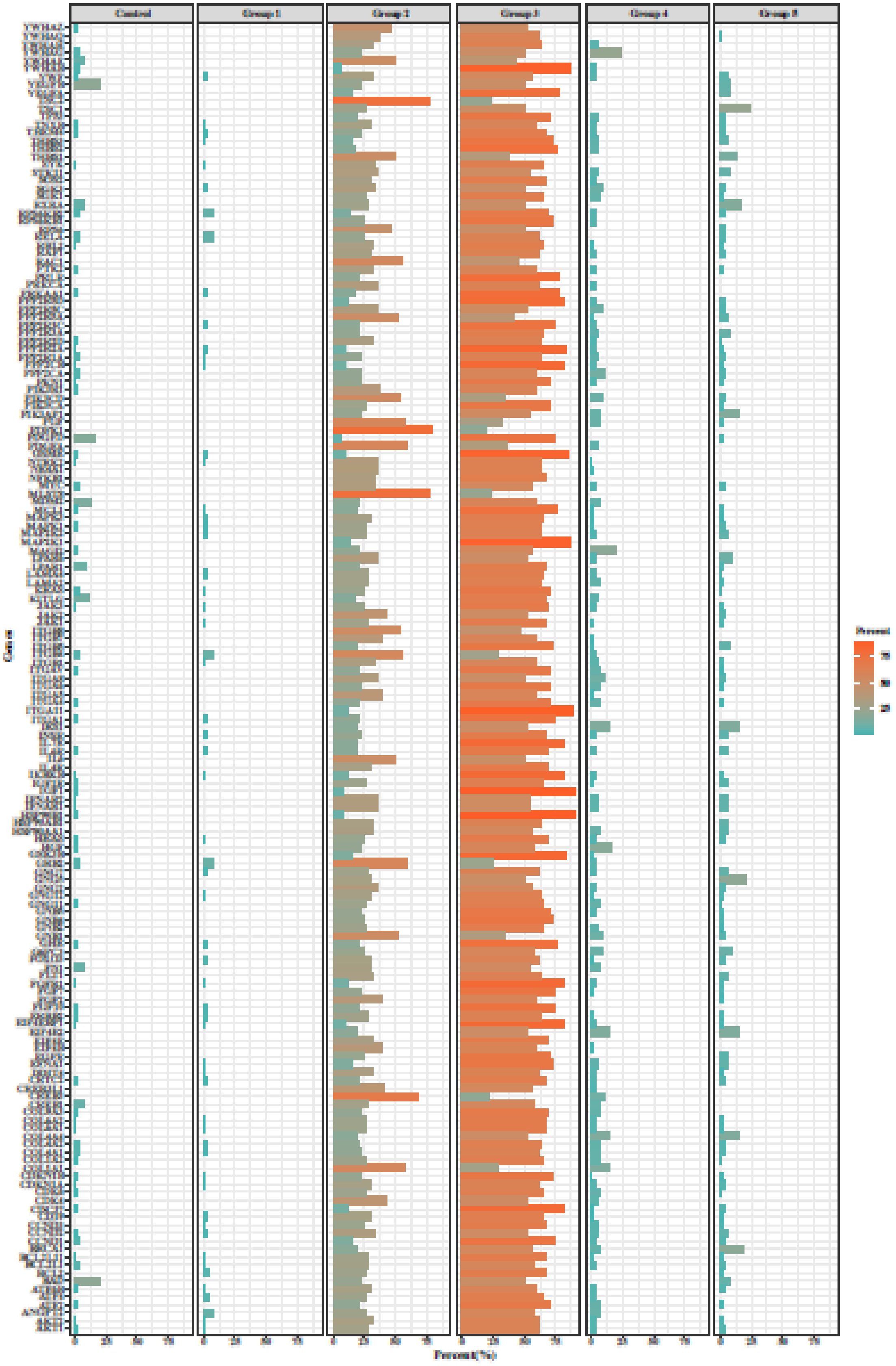

Supplementary Figure 1: Proportion of variation in PI3k-Akt signaling pathway genes in each group of cells.

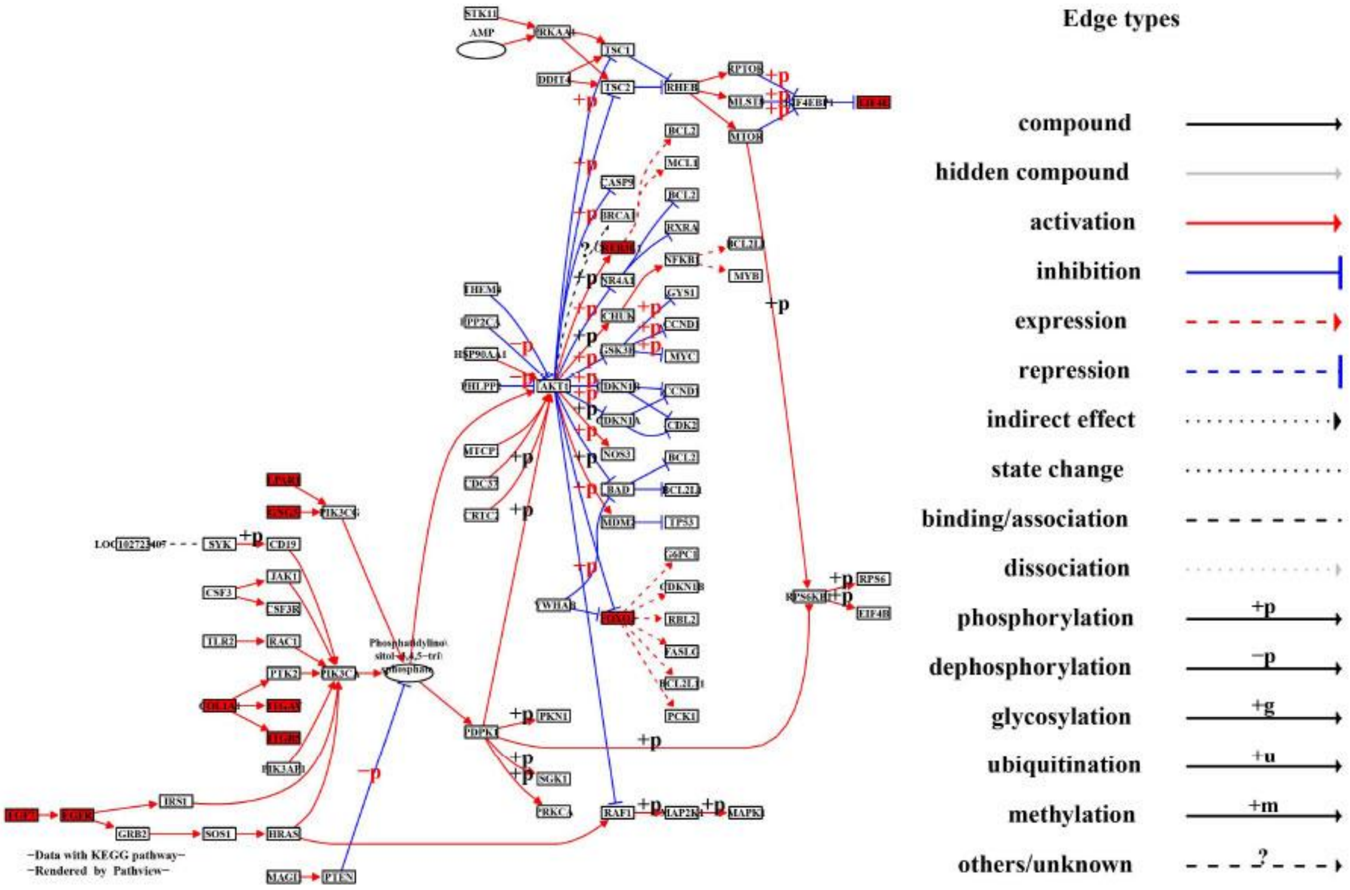

Supplementary Figure 2: PI3K-Akt signaling pathway genes with elevated expression over time are located within the PI3K-Akt signaling pathway.
